# Why Are Turkish University Students Addicted to the Internet? A Moderated Mediation Model

**DOI:** 10.3390/healthcare9080953

**Published:** 2021-07-29

**Authors:** Orhan Koçak, İlayda Yılmaz, Mustafa Z. Younis

**Affiliations:** 1 Faculty of Health Science, Istanbul University Cerrahpaşa, 34320 Istanbul, Turkey; ilaydayilmaz1996@gmail.com; 2 College of Health Sciences, Jackson State University, Jackson, MS 39217, USA; younis99@gmail.com

**Keywords:** age, internet addiction, self-esteem, university students, youth

## Abstract

Internet addiction has become a significant problem that primarily affects young people. It has an essential effect on the individual’s self-perception and assessment of their competencies. This study aimed to reveal whether there is a significant relationship between the level of internet addiction of university students and their age and self-esteem. For this purpose, internet addiction and self-esteem scales were used in addition to questions such as age, gender, the purpose of internet use, and internet daily usage time. We used a quantitative research method to obtain cross-sectional data from 400 Turkish young people using online surveys. Correlation, regression, mediation, and moderation analyses were performed using SPSS and the PROCESS macro plugin for data analysis. Internet addiction was significantly associated with self-esteem, gender, age, and daily internet usage. In addition, we discovered that self-esteem and daily usage time played a mediation role in the effect of the age variable on internet addiction. Moreover, the moderation roles of social networks, gender, and location in the impact of self-esteem on internet addiction were determined. With this study, we understood that as age increases, self-esteem triggers the decrease of internet addiction. In this sense, policies should be developed to increase self-esteem among young people to ensure the conscious use of the internet.

## 1. Introduction

Although the internet makes life easier, unnecessary and excessive use may lead to internet addiction. Widespread internet usage can rapidly turn into an addiction. Individuals find the opportunity to express their feelings and thoughts more comfortably in the virtual environment. Self-esteem, which is defined as how individuals evaluate themselves positively or negatively and the value they attribute to themselves, is higher in some individuals and lower in others. It was understood that individuals with low self-esteem prefer to spend more time on the internet instead of socializing outside [1]. Therefore, individuals’ wasting a long time on the internet can lead to internet addiction. Internet addiction also negatively affects self-worth [2], and thus individuals enter a cycle in which life is negatively affected.

The study was based on the theory developed by Mruk that low self-esteem makes individuals prone to mental illnesses such as depression, anxiety, learning difficulties, and addiction [3]. In the literature, there is an emphasis on self-esteem in the prevention and therapy of internet addiction [4,5,6,7]. This study aimed to determine how the internet’s usage affects internet addiction levels of students of different ages and to understand what role self-esteem plays in this process. While evaluating this effect, questions such as age, gender, daily internet use, place of residence, and why they use the internet were used as control variables in the study aimed at university students in Turkey. The questionnaire was prepared using the Young Internet Addiction Test-Short Form, the Rosenberg Self-Esteem Scale, and the personal information form. There is one research question in our study. This research question is RQ: “Why is internet addiction increasing among university students in Turkey?”. 

There was a significant correlation between internet addiction and self-esteem and gender, age, and regular internet use. In addition, we discovered that self-esteem mediated the association between the age variable and internet addiction. Moreover, the moderating roles of social networks, location, and gender in the effect of self-esteem on internet addiction was found. This study shows that an increasing age triggers the reduction of internet addiction due to self-esteem. In this sense, we recommend that policies be developed to increase self-esteem, especially for young people.

## 2. Theoretical Basis of the Research Hypotheses

### 2.1. Internet Addiction and Self-Esteem

The internet transformed our lives in many ways and contributed to numerous indisputably beneficial developments. However, it also resulted in undesirable effects such as internet addiction among young generations [8]. University students are more prone to be addicted to the internet than other social groups because of difficulties in their career path, gaining independence, and developing consistent relationships with friends. They can try to ignore the anxiety and stress caused by these difficulties by spending time on the internet [9]. In addition, people who have bilateral problems in real life increase their self-confidence by showing only their good qualities to the people they contact through online networks [10]. Excessive use of the internet, which develops for different reasons, triggers success, happiness, and expectation in users. As a result, users can spend more time on the internet to meet their expectations, disrupting tasks in their lives [11]. People who are curious and open to new experiences tend to be addicted to the internet compared to other individuals [12]. There are some criteria for internet addiction which require psychological or pharmacological approaches [8]. According to Beard, and Young, the criteria for internet addiction are as follows [13,14]:

1. Preoccupation with internet use.
2. Using the internet for increasing periods to ensure satisfaction with the internet.
3. Unsuccessful attempts to reduce, stop, and control the time spent on the internet.
4. Unrest, aggression, or boredom in the individual when reducing, stopping, and controlling the time spent on the internet.
5. Being active on the internet for more than the specified time.

The increasing prevalence of various activities organized in the virtual environment and the ease of communication on the internet compared with real life can trigger internet addiction disease [11]. Many psychological problems arise with excessive internet use, including increased social insecurity, worthlessness, depression, helplessness, and various fear diseases [12]. Young and Rodgers found that low self-esteem and the need for approval in relationships could cause an increase in internet use [15].

Self-esteem can be defined as whether individuals consider themselves valuable and worthy of being loved and admired [16,17]. Self-esteem is a concept that continues throughout life and is learned through internalization [18,19,20]. It plays a vital role in individuals’ ability to recognize the feelings and thoughts they develop about themselves [21,22]. People with high self-esteem are academically successful and interested in research. They do not give up quickly in the face of negativities and try to overcome the situation with their self-confidence [23,24]. According to Karahan et al., individuals with low self-esteem experience some adaptation problems with their environment and deterioration in their psychological wellbeing over time [25]. These people prefer to stay away from socializing or minimize communication within the social environment [20,24].

### 2.2. The Internet Addiction in Turkish Adolescents and Its Contributors

The widespread use of computers and the internet in Turkey has caused children to meet computers at an early age, and thus the time spent on computers and the internet has increased [26]. According to the 2019 data of the Turkish Statistical Institute (TUIK), internet use is at 79% among individuals in the 16–74 age group. Internet usage rates are 84.7% for men in the 16–74 age group, while they are 73.3% for women [27]. When the 2020 data of the Turkish Statistical Institute is evaluated for the internet usage rates by age, the age range of those who use the internet the most is 16–24, followed by the 25–34 age range. Thus, it is seen that the rate of internet usage decreases gradually as age increases in Turkey [27,28].

Changes in the lifestyle of families living in urban areas in the last two decades, the disappearance of traditional playgrounds in neighborhoods, family members focusing on economic problems, developments in mass communication, and media technologies prevent family members from spending quality time with each other. Therefore, young people are turning to the internet as an alternative [29,30]. The rapid increase in digitization rates in public, private, and voluntary sectors has made digital activity indispensable in the lives of individuals. In particular, the use of the internet is increasing rapidly among young people due to the widespread use of social media in communication and socialization as a new platform and its convenience in education and access to information [31,32].

According to Ceyhan’s research, university students who use the internet for enjoyment and to create social relationships with strangers have significantly higher problematic internet usage levels than students who use the internet to gain information [33]. A positive and low-level significant relationship was found between students’ internet use locations, internet usage frequencies, and internet addictions in a study done by Tuncalp [34]. Adolescent internet addiction was found to be positively connected with smoking, psychological problems, computer time, smartphone usage time, and playing digital games [35,36,37,38,39]. Playing games on the internet has become one of the important entertainment tools which triggers internet use in Turkey [40]. The students’ most preferred game types were action, sports, intelligence, adventure games, and the least were role-playing, educational games, and platform games [41]. As long as playing games is kept under control, it can positively affect students, providing fun, stress relief, relaxation, and socializing with people from different periods of life. Otherwise, adverse effects on young people arise when gaming becomes excessive and addictive [42].

In one study, 500 participants between the ages of 18–29 were reached, and it was determined that the internet addiction of young people was moderate. In addition, nearly half of the youth mentioned that they neglect their families, spend more and more time on the internet, and their school studies suffer because of their time on the internet [29]. In a study by Doğan and Fuat, they determined the negative effects of problematic internet use behaviors on Turkish students. Those are an inefficient use of time, negative effects on social skills and relationships, social isolation, bullying, exposure to hate speech, aggressive behaviors, learning wrong values, health problems, decrease in academic success and performance, academic procrastination behavior, and not doing homework [43]. A study conducted in Turkey discovered that playing games on the internet for an extended period can have many negative effects on the individual and social context, such as decreasing time spent with family or friends, delaying priorities and responsibilities, the emergence of health problems with prolonged inactivity, and addiction to the virtual environment by moving away from real life [40,41].

### 2.3. Self Esteem, Daily Internet Usage, and Internet Addiction

Koch and Pratelli found that individuals with low self-esteem spend more time on the internet, leading to addiction [4]. Armstrong et al. support this finding by discovering that individuals with low self-esteem spend more time on the internet, and self-esteem is an important factor in determining addiction [5]. Niemz et al. similarly stated that individuals with low self-esteem have problematic internet use [44]. When the literature was examined, many studies found that as self-esteem increases, internet addiction decreases [6,7,45,46]. In this sense, current literature shows that internet addiction and self-esteem are highly correlated with each other.

The daily internet usage rate is increasing over time as digital technologies penetrate increasingly into our lives. However, the use of the internet for educational purposes has become widespread during the COVID-19 pandemic period [47]. Therefore, young people’s use of the internet for educational purposes sometimes leads them to use it for other purposes, such as playing online games and socializing [42]. Similarly, it is also seen that young people become internet or online game addicts over time under the pretext of study [48,49]. Therefore, young people can be constantly connected to the internet via their smartphones, regardless of their purposes, such as study, play, or socialization. Hence the intertwined issues [50] may lead to intertwined internet usage purposes of young people which gradually increases their daily internet usage rates [51,52]. Ultimately, the increasing daily use of the internet, an integral part of young people’s lives, causes internet addiction. In this process, the transfer of daily life issues, such as career, socialization, psychological problems, studies, games, and entertainment, which are intertwined in the lives of young people, to the online environment is also effective [53,54,55,56,57].

### 2.4. The Direct, Mediation, and Moderation Analyses between Age and Internet Addiction

Self-esteem varies according to specific age periods throughout human life. At the beginning of primary school, self-assessment, feedback from the social environment, and self-comparison with other individuals lead to a decline in self-esteem [58]. With the advancement of age, the replacement of identity confusion in adolescence with identity formation plays an essential role in the development of self-esteem [19,59]. There is an increase in self-esteem in adulthood because specific responsibilities that fall on individuals in work–family life contribute positively to self-esteem. On the other hand, there is a sudden decrease in self-esteem in advanced adulthood because of the loss of many lifelong roles during this period, insufficient coping with stress, and loneliness [58,60]. Therefore, it was understood, up to a certain period in human life, responsibilities increase with increasing age, contributing positively to self-esteem.

There is a negative relationship between age and the duration of internet use [61,62,63]. Studies in the literature found a negative correlation between age and internet addiction [64,65]. Today’s young people have more demand to access digital opportunities. Therefore, smartphones in particular, which can lead to internet or smartphone addiction, are relatively widespread among young people in Turkey [66,67,68,69,70]. Similarly, a robust correlation was found between digital natives growing up in the cyber age and internet addiction in the USA [71]. Internet addiction is more common in young people than older adults [72]. The use of social media platforms at an early age causes young people to use the internet more [73]. Studies conducted with young people found that more than 90% of them use social media, and they log in to their accounts several times a day [74,75]. However, in recent years, it has been observed that internet use among the elderly has increased [76,77]. The middle aged and above use the internet for different reasons than young people [78]. Although not as much as young people, internet usage rates of the elderly are above the expected [79]. The internet usage rates of the elderly will likely increase gradually.

A large-scale study in 48 countries found age-related increases in self-esteem from late adolescence to middle adulthood, and males had higher self-esteem than females [19,80]. In another study conducted by O’Malley and Bachman, there was a positive correlation between self-esteem and age [81]. There is a positive link between everyday internet use and internet addiction. It was found that internet addiction increases as the length of internet usage increases [82,83,84]. It was determined in the literature that there are differences according to gender in the relationship between internet addiction and self-esteem [7]. Mo et al. found in their studies that the association between internet addiction and self-esteem was stronger among males [85,86,87]. Mamun et al. and Younes et al. found that internet addiction was higher among males in their studies [59,60]. In addition, in another study, Mo et al. and Hossain et al. found that internet addiction was more common among women [58,61]. Khan et al. found that internet addiction was similar in men and women [88].

In the studies conducted in the literature, it is understood that there are relations between the use of social network sites and applications and internet addiction. By searching, finding, and sending any text or verbal messages, videos, or images, social networks provide instant communication with only one click [89]. Multipurpose use of social networks also triggers internet addiction [90,91]. Despite being utilized for game playing and even intimate relationship purposes, social networks are an online activity in which messaging or emailing is the most popular habit [89]. Thanks to the links provided from social networks, intensive use leads to other negativities such as game addiction in individuals [92]. Many studies show that those who use social networks have more internet addiction [75,93,94]. In the study of Çam and İşbulan, it was understood that the usage of social networks is higher in men than in women [75].

It was seen that internet addiction differs according to the locations of individuals. It was understood that those living in big cities have more internet addictions than those living in small cities and rural areas [95,96]. Individuals in rural areas may have less opportunities to learn about the negative implications of excessive internet use and the need to limit internet-related activities [77]. Adolescents in urban areas are more likely than those in rural areas to engage in internet pornography, play computer games, disclose personal information to unknown individuals encountered on the internet, and to use Instant Messaging (IM), emails, and social networking services [95]. Therefore, several studies found that people who live in an urban area have higher internet addiction [91,96,97,98], whereas others found internet addiction is more prevalent in rural areas [99]. However, some studies report no variations between individuals at risk of internet addiction residing in rural areas, towns, and large cities [100,101,102,103]. In the light of the current literature, the hypotheses of the research were formed as follows:

**Hypothesis** **1** **(H1).** *Self-esteem mediates the effect of age on internet addiction.*

**Hypothesis** **2** **(H2).** *Daily internet usage mediates the effect of age on internet addiction.*

**Hypothesis** **3** **(H3).** *Gender (i), social networks (ii), and location (iii) moderate the effect of self-esteem on internet addiction.*

### 2.5. The Context of This Study

**Figure 1:**
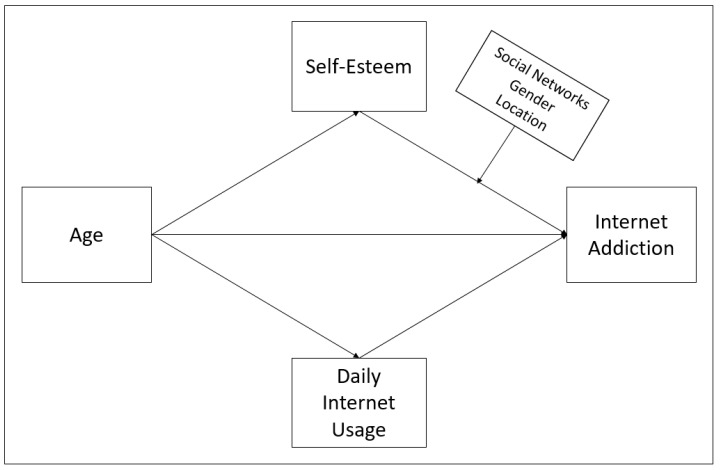
Conceptual model of research.

The use of the internet is increasing rapidly due to its beneficial contributions to all areas of life. However, uncontrolled use leads to addiction by disrupting the daily life order. Therefore, internet addiction has become a common problem in Turkey, and alternative approaches should be put forward to cope with it. While it is more common in young people, it usually decreases with aging. However, the determination of the change of this effect of aging on self-esteem and internet daily usage differentiates this study. We used age as an independent variable, online addiction as a dependent variable, and daily internet usage as a mediating variable in this study. We predicted that age has a negative mediating effect in the connection between internet addiction and self-esteem and that daily internet usage has a positive mediating role. To that goal, Figure 1 depicts first the correlation between age and internet addiction, then the mediating effects of mediator variables in the association between independent and dependent variables, and finally the moderating impacts of moderator variables.

## 3. Method

### 3.1. Study Design, Participants, and Procedure

Descriptive, correlational, and causal comparison models are used in a study conducted within quantitative research. The descriptive model investigates the current situation in a particular subject and reveals it as it is. The correlational model examines the existence of the relationship between at least two variables [104]. In our study, descriptive and correlation analyzes were performed, and a conceptual model was established, as illustrated in Figure 1. According to this model, we assumed that self-esteem had a mediating effect between age and internet addiction. In addition, as shown in Figure 1, we analyzed that connecting to social networks, gender, and location had a moderating impact on the effect of self-esteem on internet addiction.

Since this research attempted to consider a specific time reflection among people in Turkey, it was planned as a cross-sectional study. We used purposive sampling to evaluate the sample and used the survey as a medium for collecting data. The survey was conducted online between 15 March 2020, and 10 April 2020, using Google Forms. A quantitative and correlation design was used in this study. The aim was not to generalize the levels of variables, but rather to evaluate the relationship between variables and the intensity of these relationships.

The study’s universe consists of university students between 18–39 years of age and using the internet. The study sample consists of individuals willing to participate in the research in the universe. In total, 409 individuals participated in the study. However, the data collected from 9 people were excluded due to inconsistency. We asked personal information and scales questions to the participants on the internet.

### 3.2. Measures

#### 3.2.1. Personal Information Form

The form consisted of demographic questions including gender, age, class, education, living with family, and daily internet usage. The answers to the location question were reassessed as a dichotomous variable. The villages and towns were one group (coded 0) and the city and the metropolitan city the other (coded 1). The responses to the question of internet usage reasons were split into two groups. Two dichotomous variables were created for homework and others (coded 1-0) and social networks and others (coded 1-0).

In our study, demographic questions were used as control factors, but age was used as the independent variable. However, among other categorical demographic questions, the age variable was asked as an open-ended question. As a result, the age variable was employed as the continuous variable in the model.

#### 3.2.2. Young’s Internet Addiction Test-Short Form (YIAT-SF)

The 5-point Likert-type scale developed by Young and Rodgers [15] was short-formed by Pawlikowski et al. [105] and includes 12 items. A Turkish validity and reliability study was conducted by Kutlu et al. [54]. Each item is calculated over a score between 1 and 5 (1 = never, 5 = always). There is no reverse-coded item on the scale. The lowest and highest scores that can be obtained from the scale are 12 and 60, respectively. A high total score means that internet addiction is high. According to our research data, it was determined that the reliability of Young’s internet addiction is high (Cronbach’s Alpha = 0.898).

#### 3.2.3. Rosenberg Self-Esteem Scale (RSES)

The scale developed by Rosenberg [106] consists of 63 items and 12 sub-factors. Since the first ten items measure self-esteem, only this part was used in the study. A reliability study of the scale was conducted on 5024 high school students in the USA. The Turkish validity and reliability study was conducted by Çuhadaroğlu [107] on 205 high school students. The lowest and highest scores obtained from the 4-point Likert-type scale are 10 and 40, respectively (1 = very wrong, 4 = very correct). Items 3, 5, 8, 9 and 10 of the scale were scored inversely; 10–20 points indicate low self-esteem, 20–30 points is moderate, and 30–40 points is high self-esteem. According to our data, the Rosenberg self-esteem scale’s reliability was determined as high (Cronbach’s Alpha = 0.906).

### 3.3. Data Analysis

The research was designed using a quantitative method. The survey was conducted on the internet via Google Forms. After collecting the data, it was cleaned and arranged in the MS Excel program, and then the IBM SPSS version 22.0 statistics (IBM, Armonk, NY, USA) [108] package was used to analyze the research data. Demographic information of the participants was shown by percentage and frequency analysis. Correlation analysis was performed using the Pearson coefficient to determine the relationship between internet addiction, self-esteem, daily internet usage, and sociodemographic variables. Multiple regression analysis was conducted to determine the direct effects of age and other sociodemographic variables on self-esteem (Step 1), daily internet usage (Step 2), and internet addiction (Step 3). Indirect and moderation analyzes were performed to test the hypotheses. We tested the mediation and moderation hypotheses using the PROCESS macro plugin [109] and conducted simple slope tests for two-way interactions [110]. Model 14 in PROCESS macro was used for moderated mediation analyzes [109]. The significance level was set at 0.05.

## 4. Findings

### 4.1. Descriptive Analyses

In Table 1, 66.3% of the participants were women, and 33.8% were men; 56.3% of them were between 18–21, 29.5% were between 22–25, 7% were in the 26–29 age range, and 7.2% were 30 and over; 3.3% preparatory, 23.5% the first year, 15.3% the second year, 14.2% third year, 15.5% fourth year and 28% had a graduate or higher level of education. A village or town was residence to 20.3% of the participants, while a city or metropolitan city was 79.8%. According to the participants’ daily internet usage, 1% use the internet for less than 1 h a day, 15.8% for 1–2 h, 48.8% for 3–4 h, and 34.5% for 5 h or more per day. The internet was used by 6.3% for games and entertainment, 60% for social networking, 15.5% for homework and study, and 18.3% for watching movies or videos.

### 4.2. Correlation Analyses

Correlations between variables are shown in Table 2. First, the means and standard deviations of the variables are shown. According to Table 2, a statistically positive significant relationship was found between the participants’ age and self-esteem (r = 0.13, *p* < 0.01). In other words, as age increases, self-esteem levels also increase. A statistically significant negative correlation was found between the participants’ age and internet addiction (r = −0.10, *p* < 0.05). In this sense, as age increases, internet addiction decreases. Therefore, we found that as age increases, self-esteem increases, and internet addiction decreases.

A weak statistically negative correlation (r = −0.12, *p* < 0.05) was found between the participants’ daily internet usage and self-esteem. A statistically significant positive correlation was found between daily internet usage and internet addiction (r = 0.39, *p* < 0.01). In other words, there is a negative association between daily internet usage and self-esteem and a positive correlation between internet addiction and daily internet usage. According to Table 2, a moderately statistically significant negative relationship was found between the participants’ self-esteem and internet addiction (r = −0.43, *p* < 0.01). In other words, as the self-esteem of the participants’ increases, internet addiction rates decrease.

### 4.3. Regression Analyses

Multiple regression analysis results are shown in Table 3. In Table 3, Step 1, we evaluated the effect of sociodemographic variables, including age, on self-esteem. Accordingly, as age increased, self-esteem increased (B = 0.16, *p* < 0.05). Self-esteem declined as gender increased (B = −1.28, *p* < 0.05). There was no significant effect between location and self-esteem. Table 3 shows the impact of sociodemographic variables on daily internet usage in Step 2. As a result, as students aged, their daily internet usage increased (B = 0.02, *p* < 0.05). Daily internet usage was higher for males than females (B = 0.18, *p* < 0.05). Those students who were using the internet for homework had lower daily internet usage than others (B = −0.38, *p<* 0.01).

We included self-esteem and daily internet usage as predictors alongside existing sociodemographic variables in Table 3, Step 3, and the age variable had a different impact than in Step 2. Accordingly, there was no significant impact of age on internet addiction because the effect of age on internet addiction decreased and became meaningless when self-esteem and daily internet usage were included in Step 3. Males were more addicted to the internet than females (B = 2.43, *p* < 0.01). Internet addiction increased as daily internet use increased (B = 3.59, *p* < 0.001). When self-esteem improved, so did internet addiction (B = −0.50, *p* < 0.001).

### 4.4. Mediation Analyses

It was observed that the age variable, which was the independent variable in our model, had a significant effect on the dependent variables of self-esteem and daily internet usage in Table 3 and Steps 1 and 2. When self-esteem and daily internet usage were included in the analysis in Table 3 and Step 3, it was understood that the age variable did not significantly affect internet addiction.

The age variable continued its effect on the internet addiction dependent variable through the mediator variables of self-esteem and internet daily usage, as seen in Table 4. Daily internet usage had a full, positive, and significant effect as a mediator in the effect of age on internet addiction (γ = 0.0829, SE = 0.0394, 95% CI [0.0109, 0.1642]). In addition, self-esteem had a full, negative, and significant effect as a mediator in the effect of age on internet addiction (γ =−0.0893, SE = −0.0372, 95% CI [−0.1641, −0.0153]). As a result of the impact of self-esteem and daily internet usage as mediators, the direct effect of age on internet addiction was not significant (Step 3, *p* = 0.39). According to the results, hypotheses H1 and H2 were accepted.

### 4.5. Moderation Analyses

**Figure 2:**
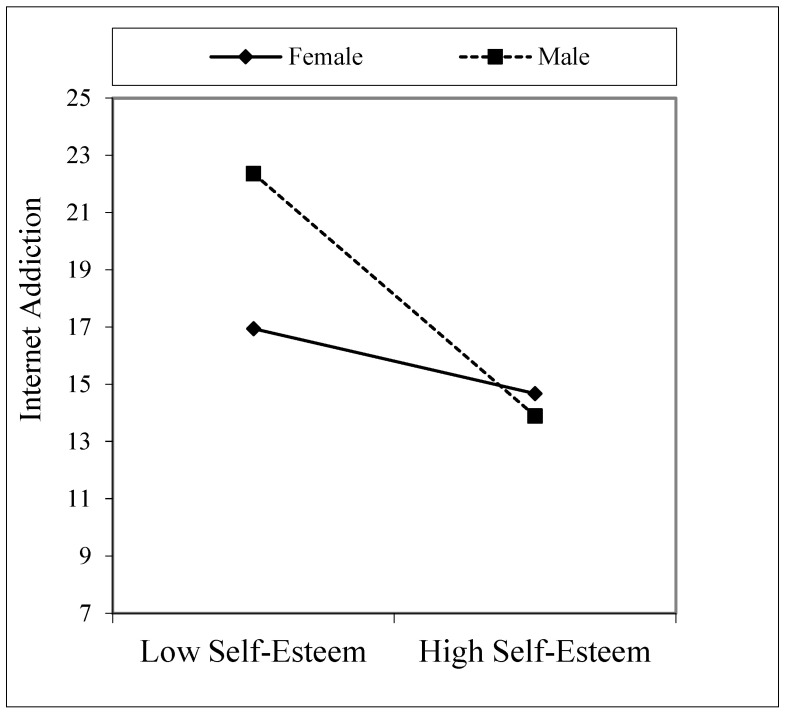
Interaction effect of self-esteem and gender on internet addiction.

Moderation analyses are shown in Table 5 in three different models, as Model 1, 2, and 3. According to the results hypothesis, H3 was accepted. Table 5, Model 1, shows the existing sociodemographic variables, self-esteem, and gender interaction variables. The effect of the self-esteem X gender interaction variable on internet addiction was significant (B = −3.02, *p* < 0.001). According to this result, there were differences in the effect of self-esteem on internet addiction according to gender. As seen in Figure 2, as self-esteem increased, males’ internet addiction levels decreased faster than females’.

**Figure 3:**
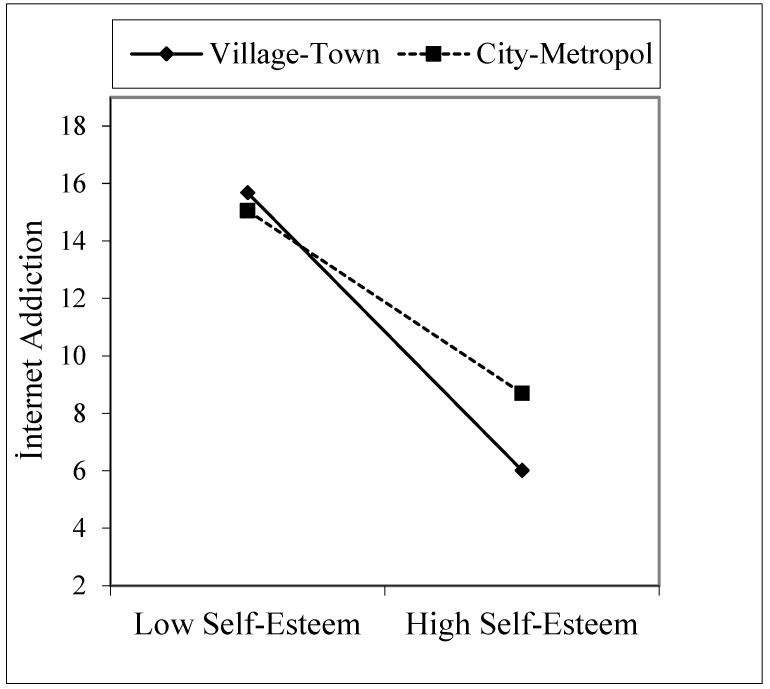
Interaction effect of self-esteem and location on internet addiction.

Table 5, Model 2 shows that the interaction variable of self-esteem and location was included in the existing sociodemographic variables. The effect of the self-esteem X location interaction variable on internet addiction was significant (B = 3.69, *p* < 0.001). Accordingly, there was a statistically significant difference in the effect of self-esteem on internet addiction between village/town and city/metropolitan residents. According to the graphic depicted in Figure 3, as self-esteem increased, the level of internet addiction decreased faster in those living in a village/town area than those living in a metropolitan area.

**Figure 4:**
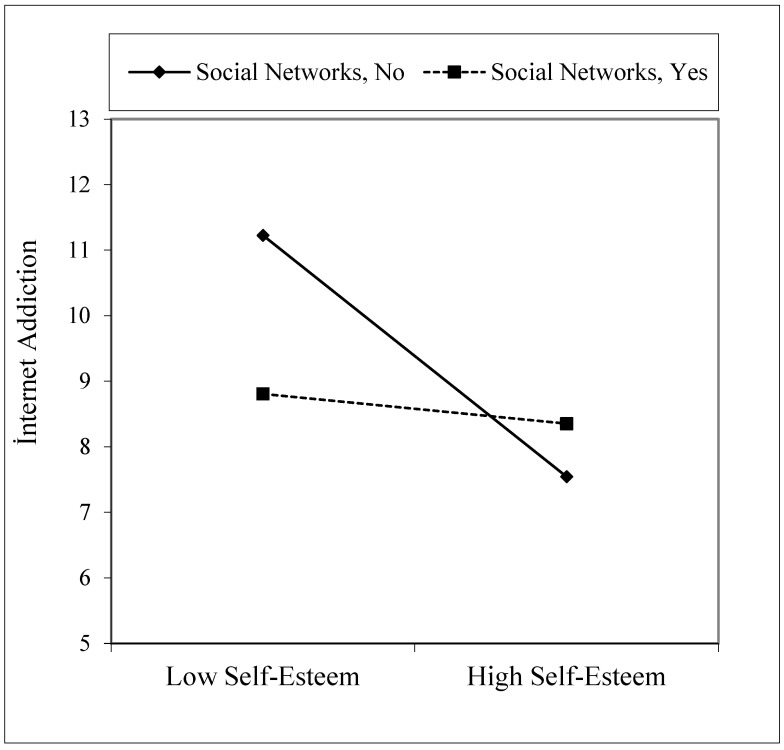
Interaction effect of self-esteem and social networks on internet addiction.

As seen in Table 5, Model 3, we found the effect of self-esteem X for social networks interaction variable on the internet addiction dependent variable to be significant (B = 1.86, *p* < 0.05). Accordingly, there was a statistically significant difference in the effect of self-esteem on internet addiction between those who use the internet to reach social networks and those who use it for other purposes. As shown in Figure 4, it was understood that as the self-esteem of those who use the internet to reach social networks increased, internet addiction decreased much slower than those who use it for other purposes. In other words, while the self-esteem of those who use the internet for social networks increases, internet addiction becomes more resistant to decreasing compared with those who use it for other purposes.

### 4.6. Results of the Research Model

**Figure 5:**
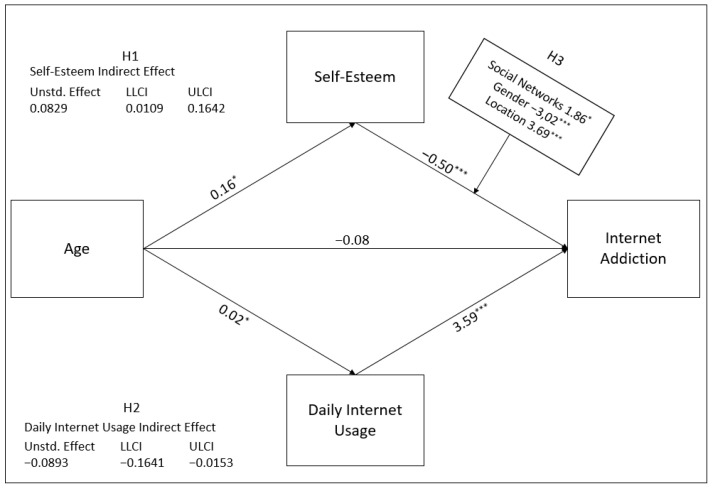
Results of the proposed research model, * *p* < 0.05, *** *p* < 0.001.

After performing the direct, indirect, and moderation analysis of the conceptual research model in Figure 2, the results are shown in Figure 5 below. The coefficients giving the direct effect between each factor are shown between those two factors. The H1 hypothesis shows the mediating effect of self-esteem in the effect of age on internet addiction. The H2 hypothesis shows the mediating effect of daily internet usage in the effect of age on internet addiction. The H3 hypothesis shows the moderation effects of social networks, gender, and location in the effect of self-esteem on internet addiction.

## 5. Discussion

We tried to understand the mediation of self-esteem and daily internet usage in the association between age and internet addiction within the study. Additionally, the moderation roles of gender, social networks, and location in this relationship were examined. As a result of the analyzes, the hypotheses, H1, H2, and H3 were accepted. Sociodemographic data were used as control variables while examining the relationship between independent and dependent variables. We found that as the self-esteem levels of the participants increased, their internet addiction levels decreased significantly. Similarly, some studies revealed an inverse relationship between internet addiction and self-esteem [24,79,111,112]. When the literature was examined, we found studies in which internet addiction decreases as self-esteem increases [6,7,45,46]. This situation shows that the two variables are highly correlated with each other. With the increase in self-esteem, there is an increase in the positive emotions attributed to the individual. When the studies on the relationship between internet addiction and self-esteem were examined, Koch and Pratelli stated that individuals with low self-esteem spend more time on the internet and reach addiction [4]. Armstrong et al. supported this finding and revealed that individuals with low self-esteem use the internet more, and self-esteem is an essential factor in determining addiction criteria [5]. Niemz et al. similarly stated that individuals with low self-esteem have problematic internet use [44]. Berardis et al. found that adolescents’ low self-esteem could cause internet addiction [113]. Contrary to these studies, Yıldız found a positive relationship between self-esteem and internet addiction in a study conducted with 200 university students in Istanbul [114]. Eroğlu and Bayraktar could not find a meaningful relationship between self-esteem and internet addiction in their research with 120 people living in Istanbul [115]. We understood from studies that individuals with low self-esteem try to meet their needs for being accepted and valued by society over the internet.

We found that males had higher internet addiction levels than females. There were studies by Balta and Barış, Ata et al., Kuzucu et al., and Sargın supporting this finding [116,117,118,119]. Wu et al. stated that female students’ internet addiction rates were higher than males’ [120]. Contrary to these findings, no significant relationship was found between gender and internet addiction in the works of Çelik & Odacı, Zorbaz and Dost, Yıldız, and Yaygır [24,114,121,122]. The emergence of various findings in the studies can be interpreted as a result of the variability of the sample groups and the influence of gender by other demographic variables. When self-esteem and gender were examined, we found that females’ self-esteem levels were higher than males’. Özkan’s study supported this finding [123], whereas Dikici, Yıldız, and Otacıoğlu did not find a significant relationship between gender and self-esteem in their studies [114,124,125].

When the relationship between internet addiction and daily internet use was examined, we found that individuals who use the internet for 5 h or more per day had higher addiction levels. Likewise, Yaygır revealed that people who spend 12 h or more on the internet every day [24], and Gokcearslan and Gunbatar found that people who spend 3 h or more on the internet every day, have a higher degree of internet addiction [126]. Therefore, the time spent on the internet can be considered an essential factor in addiction formation.

We found that individuals who use the internet for social networks and games scored higher internet addiction levels than those who use the internet for other reasons. Balcı and Gülnar found that individuals who use internet games had higher addiction levels [127]. However, individuals who used the internet for homework or research purposes had higher self-esteem and lower internet addiction, supported by Koyuncu et al. [128] and Şaşmaz et al. [129]. In our study, there was a statistically significant difference in the effect of self-esteem on internet addiction between individuals who used the internet to access social networks and those who did not. Although there is no moderation analysis directly related to social networks in the literature, studies indicate that social networks trigger or increase internet addiction [91,93]. The use of social networks provides many activities that individuals need and increases their dependence and ultimately their internet addiction [75,89,94,130]. According to Yaygır, individuals who use the internet for shopping purposes had higher levels of internet addiction and could not find a significant relationship between the reasons of internet usage and self-esteem [24]. Yıldız was unable to identify a meaningful relationship between the purpose of using the internet and internet addiction and self-esteem in the study. Young people need more socialization than older people. Connecting to social networks is one of the more common reasons for using the internet to satisfy this need.

According to the study, a positive correlation was found between those living in big cities and internet addiction. Similar results were found in the literature [91,95,96,97,98]. In this sense, the use of the internet in cities for purposes such as emails, social networking services, shopping, work, etc., increases addiction to the internet [95]. On the contrary, some studies found no difference between rural and urban areas regarding internet addiction [100,101,102,103].

Some studies could not find a meaningful relationship between age and self-esteem [131,132]. However, we found a positive direct relationship between age and self-esteem. Studies show that self-esteem increases with age [59,133,134]. In most of this research, it was discovered that self-esteem began to rise steadily during and after adolescence. Similar to this research’s findings, studies showed that internet addiction declines as age increases [78,120,135]. However, Aslan and Yazıcı, and Taş were unable to identify a significant relationship between age and internet addiction [136,137]. It was thought that the sample group and other sociodemographic variables affected the differentiation of the findings.

When the effect of age on internet addiction was examined, we determined that self-esteem played a mediation role. As the age increased, self-esteem increased, and internet addiction decreased. This situation can be interpreted due to a decrease in dependence on the family and the environment with the advancement of age, ending the search for identity, self-value development, having roles in society, and participation in working life. With the introduction of technology into people’s daily lives with smartphones, tablets, and computers in business and private lives (as an integral part), the internet has gained more importance. The fact that the age group, which is defined as generation Z, has access to the internet and prefers to socialize on the internet has caused internet addiction. Therefore, as age progresses, both the differences between generations and the differences in self-esteem will become essential in decreasing internet addiction.

We generated one research question. This research question is RQ: “Why is internet addiction increasing among university students in Turkey?” Internet addiction is a type of behavioral addiction that can cause psychological and physical health problems in individuals. In particular, the internet, which children start to use at a very young age, causes addiction with high usage rates among university students. Moreover, the number of applicants for internet addiction in psychiatry clinics is increasing. Therefore, it poses a severe risk for the future of Turkish society.

Since internet addiction is a behavioral disorder, first of all, young people’s behaviors towards using the internet should be changed. For this purpose, we tried to understand the effect of self-esteem, which increases with age, on internet addiction. In this study, we found that self-esteem had a significant effect on reducing internet addiction. Self-esteem develops with increasing responsibilities along with individuals’ attitudes towards work, education, career, family life, and values. Since these responsibilities are more common in middle age, self-esteem is high in the middle age period. Therefore, it can be ensured that young people have these responsibilities at an earlier age by providing education both in the family and at school.

The fact that young people are in frequent contact with the internet due to their smartphone use is another critical issue that increases internet addiction. Our study found that the increase in daily use of the internet also triggers internet addiction among young people. The internet has become an integral part of life and has an innumerable facilitating contribution. Online digital technologies are intertwined in every field, and it is not possible to give up easily. Therefore, it is essential to avoid unnecessary use of the internet, which negatively affects daily life. For this purpose, approaches should be developed to reduce the unnecessary internet usage time of young people and highlight beneficial internet use. In this sense, our study determined that the internet addiction of those who use the internet for homework is significantly lower than those who use it for other purposes. In addition, it was determined that the self-esteem levels of the young people who use the internet for homework were higher than those who use the internet for other reasons. Moreover, it was found that those who use the internet for homework had less daily internet usage than those who use the internet for other reasons.

Internet addiction is moving towards a dimension that threatens the future of societies through young people. As can be understood from our study, self-esteem, which increases with the responsibilities of individuals, plays an essential role in reducing internet addiction. In our research, young people who use the internet for homework use the internet less daily, have high self-esteem, and have a low internet addiction, confirming the cycle of responsibility, self-esteem, and addiction. Therefore, there is a need for policies and practices that will increase the self-esteem of young people. In this sense, self-esteem should be seen as a value that young people can strengthen themselves. In other words, some policies which will contribute to self-esteem, which already exists in middle and advanced ages, should be produced in education, family, and social fields for young people.

## 6. Limitations and Suggestions for Future Studies

This study was a quantitative study that measured the relationship between university students’ age, daily internet usage, internet addiction levels, their self-esteem levels, and the variables that affect this relationship. The exclusion of qualitative methods (observation, interview, etc.) from the study’s scope constitutes the limitation of the study as the questions for measurement were only delivered to the participants via Google Forms on the internet. Another limitation of the study was that the sample consists of different age groups who are continuing their university education. Therefore, examining new studies using different samples and especially a more comprehensive age range will compare the similarities and differences between different groups. In the future, it is necessary to carry out studies that focus on different groups and that show comparisons between groups and periods to reach more specific findings.

## 7. Conclusions and Some Implications

When the research findings were examined, it was determined that there is a negative relationship between internet addiction and self-esteem. Among the demographic variables, gender, age, and internet daily usage significantly affect internet addiction and self-esteem. In addition, it was concluded that self-esteem and daily internet usage play the role of mediation in the impact of the age variable on internet addiction. That is, as age increases, self-esteem increases, and internet addiction decreases. Moreover, daily internet usage has a positive impact on the association between age and internet addiction. It was determined that gender, location, and social networks as the internet usage reason play a moderation role in the effect of self-esteem on internet addiction. This situation reveals the determining role of subjective factors and factors related to internet usage in becoming addicted to the internet. Likewise, self-esteem varies, being affected by both factors. It was understood that using the internet to spend time on social networks for a long time creates addiction. Contrary to this result, using the internet for less time and educational purposes does not make an addiction. It was observed that male students and students who are still in their first years of university are at higher risk of being addicted to the internet.

It is known that directing children to sport or artistic activities from elementary school and supporting their success plays a vital role in forming self-esteem and decreasing daily internet usage. In this context, educators and families have more responsibility. Notably, young people’s presence as participatory and social individuals in society will contribute to healthy spiritual development and self-esteem. Individuals with high self-esteem will behave pragmatically, and, instead of developing internet addiction, will use the internet correctly and as required for their benefit. Therefore, sports activities, values, and personality training that will increase self-esteem and decrease daily internet usage can support young people. Additionally, it is necessary to raise awareness of internet addiction, create various public spots, and provide training to students and their families on conscious internet use in decreasing the internet’s negative effect.

Conducting more comprehensive studies to reveal the relationship between internet addiction and different variables such as school, family structure, peers, and parental attitudes, which are thought to be related to self-esteem that start to develop from childhood, will enrich the literature.

## Figures and Tables

**Table 1:** Descriptive statistics.

Variables	Categories	*Frequency*	%	Mean	Std. Dev.
Gender					
	Female	265	66.3		
	Male	135	33.8		
Age				22.12	4.112
Class					
	Prep.	13	3.3		
	1. Class	94	23.5		
	2. Class	61	15.3		
	3. Class	57	14.2		
	4. Class	62	15.5		
	Master	113	28.2		
Location (0–1)					
	Village/Town	81	20.3		
	City/Metropolitan city	319	79.8		
Daily Internet Usage					
	Less than 1 h	4	1		
	1–2 h	63	15.8		
	3–4 h	195	48.8		
	5 h and more	138	34.5		
Internet Usage Reasons					
	Game/Entertainment	25	6.3		
	Social Networks	240	60		
	Access Information	62	15.5		
	Watch Film/Video	73	18.3		

**Table 2:** Means, standard deviation and correlations.

No.	Variables	Mean	Std. Dev.	1	2	3	4	5	6
1	Gender 1–2, (f–m)	1.34	0.47						
2	Age	22.12	4.11	0.03					
3	Daily Internet Usage (h)	3.17	0.72	0.13 *	0.06				
4	For Social Networks (0–1)	0.60	0.49	−0.10	−0.05	0.10 *			
5	Location (0–1)	0.80	0.40	−0.10 *	0.13 **	−0.06	0.06		
6	Self-Esteem	27.83	5.23	−0.13 **	0.13 **	−0.12 *	0.01	0.07	
7	Internet Addiction	25.46	8.22	0.24 **	−0.10 *	0.40 **	0.04	0.10 *	−0.43 **

Note. For Social Networks 0 = No, 1 = Yes; For location 0 = village/town, 1 = city/metropolitan, h = hours, * *p* < 0.05, ** *p* < 0.01, N = 400.

**Table 3:** Direct main effects on self-esteem, daily internet usage, and internet addiction.

Variable	Step 1: Self-Esteem			Step 2: Daily Internet Usage			Step 3: Internet Addiction		
	B	SE	*p*	B	SE	*p*	B	SE	*p*
(Constant)	27.54	1.90	<0.001	2.59	0.23	<0.001	28.92	3.02	<0.001
Age	0.16	0.08	0.029	0.02	0.01	0.022	−0.08	0.09	0.386
Gender 1–2, (f–m)	−1.54	0.63	0.014	0.18	0.08	0.020	2.43	0.73	0.001
For Social Networks (0–1)	0.81	0.71	0.260	0.02	0.09	0.790	−0.87	0.82	0.288
For Homework (0–1)	1.94	1.00	0.052	−0.38	0.12	0.001	−3.41	1.16	0.003
Location (0–1)	0.50	0.74	0.503	−0.08	0.09	0.363	−0.42	0.85	0.618
Daily Internet Usage							3.59	0.48	<0.001
Self-Esteem							−0.50	0.06	<0.001
F		4.08			5.09			30.46	
*p*		<0.01			<0.001			<0.001	
R2		0.05			0.06			0.352	

Note. For Homework 0 = No, 1 = Yes; For Social Networks 0 = No, 1 = Yes; For Location 0 = village/town, 1 = city/metropolitan.

**Table 4:** Total, direct, and indirect regression analysis on internet addiction.

					Unstandardized	SE	LLCI	ULCI	
Total Effect of Age on Internet Addiction					−0.1091	0.1085	−0.3223	0.1042	N. S.
Direct Effect of Age on Internet Addiction					−0.1026	0.0940	−0.2874	0.0821	N. S.
Path					Unstandardized				
Age	>	DIU	>	IA	0.0829	0.0394	0.0109	0.1642	Sig.
Age	>	Self-Esteem	>	IA	−0.0893	0.0372	−0.1641	−0.0153	Sig.

DIU = Daily Internet Usage, IA = Internet Addiction.

**Table 5:** Direct interaction effects on internet addiction.

Variable	Model 1			Model 2			Model 3		
	B	SE	*p*	B	SE	*p*	B	SE	*p*
(Constant)	10.14	5.99	0.091	46.31	4.72	<0.001	36.76	3.84	<0.001
Age	−0.11	0.09	0.231	−0.10	0.09	0.295	−0.10	0.09	0.287
Gender 1–2, (f–m)	2.40	0.77	0.002	2.09	0.78	0.007	2.53	0.78	0.001
For Homework (0–1)	−3.57	1.23	0.004	−3.62	1.23	0.003	−3.20	1.26	0.011
For Social Networks (0–1)	−1.04	0.87	0.233	−0.86	0.87	0.325	−0.88	0.88	0.321
Location (0–1)	−0.04	0.91	0.966	−0.16	0.90	0.856	−0.38	0.92	0.677
DIU	3.84	0.51	<0.001	4.02	0.51	<0.001	3.97	0.52	<0.001
SE	0.18	0.18	0.333	−1.03	0.13	<0.001	−0.72	0.09	<0.001
SE X Gender	−3.02	0.71	<0.001						
SE X Location				3.69	0.87	<0.001			
SE X Social Network							1.86	0.73	0.011
F		30.83			30.85			28.59	
*p*		<0.001			<0.001			<0.001	
R2		0.39			0.36			0.37	

Note. For Homework 0 = No, 1 = Yes; For Social Networks 0 = No, 1 = Yes; For Location 0 = village/town, 1 = city/metropolitan; DIU = Daily Internet Usage, SE = Self-Esteem.

## Data Availability

The data that support the findings of this study are available on request from the corresponding author.
